# Factors Influencing the Clinical Outcomes of Two-Stage Re-Implantation in Patients With Periprosthetic Joint Infection After Total Knee Arthroplasty

**DOI:** 10.7759/cureus.42566

**Published:** 2023-07-27

**Authors:** Ahmet Şenel, Yusuf Öztürkmen, Murat Eren, Engin Carkci, Esra Circi, Enes Kanay, İlhan Açıkgöz

**Affiliations:** 1 Orthopedics and Traumatology, Istanbul Training and Research Hospital, Istanbul, TUR

**Keywords:** knee, antibiotherapy, spacer, two-stage revision arthroplasty, periprosthetic joint infection

## Abstract

Introduction

Infection is one of the most distressing complications of total knee arthroplasty (TKA), requiring a long treatment process and may negatively affect patient satisfaction. All surgeons aim to achieve infection-free survival, painless, functional, and stable knee after treatment of periprosthetic joint infection (PJI) with two-stage revision treatment. Many factors play a role in determining clinical outcomes. We aimed to evaluate the factors influencing the clinical outcomes of patients undergoing two-stage revision knee arthroplasty for PJI.

Methods

Forty-nine patients were retrospectively evaluated. Forty-four patients met the inclusion criteria. Spacer types, growth rates in culture, types and amount of antibiotics added to the cement, and intervals between stages were evaluated. Pre- and post-treatment infection parameters, changes in the range of motion (ROM), clinical and functional (C&F) Knee Society Score (KSS) results, and complications were also studied.

Results

After a mean follow-up of 48.8 ± 16.5 months, re-infection was detected in five out of 44 patients (10.4%). No significant difference was noted regarding C&F KSS when comparing time intervals between the two stages, whether they were shorter or longer than 10 weeks. However, better ROM results were obtained in patients with less than 10 weeks between stages. The relationship between spacer type, ROM, and C&F KSS was not found to be significant. Particularly, the addition of 4g of teicoplanin to the cement shortened the time between the two stages.

Conclusion

C-reactive protein (CRP) and erythrocyte sedimentation rate (ESR) levels can be considered safe parameters for diagnosis, reimplantation timing, and follow-up. The use of dynamic spacers or reimplantation performed within 10 weeks after the first stage is associated with better ROM outcomes. Additionally, the addition of teicoplanin to the cement shortened the duration of antibiotic therapy.

## Introduction

Infection is the most distressing complication of total knee arthroplasty (TKA), requiring a lengthy treatment process and adversely affecting patient satisfaction [1-3]. While the infection rate after primary arthroplasty ranges between 0.5% and 1.9%, it increases to 10% in cases of revision knee arthroplasty. Several treatment modalities, such as the debridement, antibiotics, and implant retention (DAIR) procedure, one or two-stage revision arthroplasty, arthrodesis, and amputation, have been proposed as treatment options for periprosthetic joint infection (PJI); however, the two-stage revision surgery appears to be the most commonly preferred procedure [1]. Along with clinical findings, C-reactive protein (CRP) is one of the most sensitive parameters and is widely used in everyday clinical practice, especially for evaluating the effectiveness of medical treatment over time. Additionally, controversy exists regarding definitive threshold values [2-4]. To achieve normal CRP and erythrocyte sedimentation rate (ESR), prolonged antimicrobial therapy between the stages has been the generally accepted care for two-stage procedures. However, some have suggested that a short course of antibiotics with significant declines is just as effective [4,5]. Identifying the causative microorganism constitutes utmost importance in diagnosing and successfully managing infected knee arthroplasty and selecting appropriate antimicrobial therapy. In the literature, the rate of negative culture results varies between 0 and 25% [6]. In addition to eradicating the infection, achieving a functional knee joint is another essential outcome expected from treating infected knee arthroplasty. The use of dynamic spacers has provided many advantages in this regard [7]. All surgeons aim to achieve infection-free survival and a painless, functional, and stable knee after treating periprosthetic joint infection (PJI) with two-stage revision treatment. Several factors play a significant role in determining clinical outcomes, including the type of spacer used (dynamic or static) after the removal of implants, the specific antibiotic added to the spacer, the results of culture and/or histopathology, the duration of the prescribed antibiotic, and the proper timing of reimplantation. This study aimed to evaluate the factors influencing the clinical outcomes of patients who underwent two-stage revision knee arthroplasty with a diagnosis of PJI after TKA.

## Materials and methods

Forty-nine patients diagnosed with infected knee arthroplasty, who underwent two-stage knee revision arthroplasty at our clinic between 2011 and 2020, were retrospectively analyzed. The study included only those patients who had well-documented clinical and functional results as determined by the American Knee Society Score (KSS) System both before and after treatment. Additionally, all included patients had accessible radiological examinations and had been followed up for a minimum of 24 months. These patients also provided written informed consent for their data to be used in this study. Patients with a follow-up period of less than 24 months, bilateral TKA infection, one-stage revision knee arthroplasty, and re-infection after the previous two-stage procedure were considered as exclusion criteria. Forty-four patients met the inclusion criteria. Our Institutional Ethics Committee at the Istanbul Training and Research Hospital approved the study (Date: 02/09/2022; No: 280). The diagnosis of infection was based on the algorithm described by the Musculoskeletal Infection Society (MSIS). The diagnosis of PJI, according to the guidelines provided by MSIS, involves two major criteria: the presence of two positive periprosthetic cultures and the existence of a sinus tract communicating with the joint. Furthermore, the diagnosis of PJI can be confirmed if a minimum of three out of six minor criteria are met. These minor criteria include elevated levels of serum CRP and ESR, an increased WBC count in the synovial fluid, an elevated percentage of polymorphonuclear neutrophils in the synovial fluid, the presence of purulence in the affected joint, a positive histologic analysis of periprosthetic tissue, or a single positive culture [8]. Range of motion (ROM) and knee instability were recorded. Standard radiographs of both knees were examined comparatively. CRP, ESR, and WBC levels were evaluated along with routine tests. Joint aspiration was performed in all patients. The surgical procedures were performed by two experienced surgeons. In the first stage, the components and residual cement were removed. Approximately five or six periprosthetic tissue samples were taken for microbiological and histopathological analysis. Meticulous surgical debridement of the infected tissues was performed. Then, the gentamicin-loaded cement (AF Cement 1G, Synergie Ingeniere Medicale SARL, Chamberet, France) was prepared with different types of antibiotics (teicoplanin, imipenem) based on the culture results of preoperative knee aspirates. In our study, either 2 g or 4 g of teicoplanin was added to each 40 g bag of cement for 23 and 12 patients, respectively. The operating surgeon determined the dose of teicoplanin. In addition, 2 g of imipenem was added to each 40 g bag of cement for nine patients. Teicoplanin was generally preferred for treating Gram-positive microorganisms, while imipenem was preferred for Gram-negative microorganisms. Due to the predominant presence of Gram-positive microorganisms in culture samples at our clinic, teicoplanin was the primary choice for patients with culture-negative results. However, the surgeon made the final determination of antibiotic preference in these patients intraoperatively. Ligament laxity, large bone, and soft tissue loss in the knee were evaluated intraoperatively for the appropriate spacer decision. The first stage was completed by the placement of a non-articulating (static) spacer prepared by handcrafted cement in 21 patients and the gentamicin-containing articulating (dynamic) prefabricated spacer (Spacer K, Tecres S.p.A., Verona, Italy) in 23 patients along with the prepared cement into the joint space.

After the first stage, subsequent to the consultation with the infectious diseases specialist, culture-positive patients were administered antibiotic treatment against the pre-identified microorganism, while culture-negative patients received empirical broad-spectrum antibiotic therapy. Afterward, the antibiotic therapy was appropriately modified based on the intraoperative culture results. The patients who received adequate IV antibiotic treatment were discharged and followed up with an interval of two weeks. During the controls, the surgical area was evaluated. CRP and ESR levels were checked. Revision knee arthroplasty was planned for patients whose infection parameters returned to normal (primarily whose CRP and ESR levels remained within the normal range after cessation of antimicrobial therapy for at least two weeks) and who had no clinical evidence of infection. Five patients (11.4%), who exhibited an inadequate response to IV antibiotic treatment over six weeks, underwent repetitive debridement and antibiotic-loaded spacer exchange due to persistent infection. At the second stage, condylar constrained revision knee prosthesis (Vanguard SSK 360, Biomet, Inc., Warsaw, IN, USA) was placed in 38 (86%) patients, Triathlon total stabilizer knee system (Stryker Orthopaedics, Mahwah, NJ, USA) was implanted in three (7%) patients and a hinged modular knee revision prosthetic system (RT-Plus Modular Rotating Hinged, Smith & Nephew, USA) was used in three (7%) patients who had knee instability due to ligament failure. Isometric and progressive ROM exercises were initiated on the first postoperative day after the second stage. The patients were followed-up at months 1, 3, 6, and annually thereafter. Pre- and post-treatment C&F results were determined in accordance with the KSS [9]. Statistical analyses were performed using SPSS software version 25.0 for Windows (IBM Corp., Armonk, NY, USA). Descriptive statistics were expressed as mean, standard deviation (SD), median, minimum, and maximum values for numerical variables, and as numbers and percentages for categorical variables. For numerical variable comparisons between two independent groups, Student's t-test was employed when the data met the normal distribution condition, whereas the Mann-Whitney U test was utilized in the absence of a normal distribution. ANOVA was performed when the differences of repetitive numerical variables in the dependent groups had normal distribution, whereas the Friedman test was used when the normal distribution was not detected. A P-value of less than 0.05 was considered statistically significant.

## Results

Thirty-two female and 12 male patients with a mean age of 70.1±9.5 (range: 46-91) were followed up for an average of 48.8 ± 16.5 months (Table 1). A total of 31 patients (70.5%) were culture positive, and 13 patients were culture negative (29.5%) (Table 2). The duration of antibiotic use between the two stages was 8.1 ± 3.6 weeks (range: 3-20 weeks).

**Table 1:** Demographic data of the patients who underwent two-stage revision knee arthroplasty due to periprosthetic knee infection (n:44).

Demographic data		Mean±SD	n (%)
Mean age (years)		70.1±9.5 (range: 46-91)	
Gender	Female		32 (72.7%)
	Male		12 (27.3%)
Comorbidities	Diabetes Mellitus		14 (31.8%)
	Hypertension		10 (22.7%)
	Heart Failure		4 (9.1%)
	Chronic Obstructive Pulmonary Disease		3 (6.8%)
	Rheumatoid Arthritis		1 (2.3%)
	Chronic Renal Failure		1 (2.3%)
	None		11 (25.0%)
Symptoms	Pain		28 (63.6%)
	Infected fistula		15 (34.1%)
	Skin problem and patellar tendon rupture		1 (2.3%)
Mean time from primary knee arthroplasty to diagnosis of infection (months)	Female	31.0±28.9	
	Male	46.7±75.6	
	Total	35.3±46.0 (range: 1-240)	
Mean follow-up (months)		48.8 ± 16.5 (range:30-89)	

**Table 2:** Rate of microorganism growth in culture (n:44).

Culture results	n (%)
Culture positive	31 (70.5)
Staphylococcus epidermidis	14 (31.8)
Methicillin-resistant Staphylococcus aureus	4 (9.1)
Staphylococcus aureus	3 (6.8)
Pseudomonas aeruginosa	2 (4.5)
Enterococcus spp.	2 (4.5)
Escherichia coli	1 (2.3)
Serratia marcescens	1 (2.3)
Staphylococcus haemolyticus	1 (2.3)
Polymicrobial	1 (2.3)
Streptococcus pyogenes	1 (2.3)
Citrobacter braakii	1 (2.3)
Culture negative	13 (29.5)

The mean time from the first stage to revision surgery was 17.1±18.7 weeks (range: 4-120 weeks). The effect of the time interval between the two stages (at a limit of 10 weeks and longer than 10 weeks) on post-treatment results was also evaluated. Better results were achieved when the time interval was limited to 10 weeks. The change in ROM was statistically significant (p=0.013), but there was no statistically significant difference between the C&F KSS results (p=0.384 and p=0.366, respectively) (Table 3).

**Table 3:** The impact of a two-stage duration threshold of 10 weeks on ROM, as well as clinical and functional KSS results post-treatment. ROM: Range of motion; KSS: Knee Society Score. P-value <0.05 was considered statistically significant.

	Duration between the two stages		
	≤10 w (n:20)	>10 w (n:24)	P-value
	Mean+SD	Mean+SD	
ROM (˚)	95.3±8.8	84.7±17.4	0.013
KSS Clinical	77.9±8.3	75.0±12.6	0.384
KSS Functional	67.0±12.5	62.1±20.6	0.366

Culture-positive patients received antibiotic treatment for a shorter period of time (mean 7.7±3.0 weeks) than culture-negative patients (mean 8.9±4.9 weeks). However, the difference between the two groups was not statistically significant (p=0.427). The CRP and ESR levels that were measured before the first stage, between the two stages, and after revision are shown in Table 4. Considering these values, changes in both parameters were statistically significant at the time of diagnosis, in response to the infection treatment between the two stages, and the post-revision follow-up period (p <0.01 for both values).

**Table 4:** The change in CRP and ESR levels during treatment and the effect of pre-reimplantation and post-revision CRP and ESR levels on post-revision complications. CRP: C-reactive protein; ESR: Erythrocyte sedimentation rate. Normal CRP values are below 0-5 mg/L. Normal ESR values are below 20 mm/hr. p<0.05 was considered statistically significant.

Serum Infection Parameters	Before treatment	Before re-implantation			After treatment	P-value	
CRP (mg/L) (min-max)	15.4±3.8	1.4±2.2			2.1±4.6	<0.001	
	(11.3-28.0)	(0.1-14.0)			(0.2-31.0)		
ESR (mm/h) (min-max)	73,5±24.5	37,3±19,6			36.2±23.1	<0.001	
	(33-116)	(3-85)			(7-95)		
Pre-reimplantation levels			Post-revision complications				
			Yes (n:8)	No (n:36)			P-value
			Mean+SD	Mean+SD			
CRP (mg/L)			2.8±4.6	1.1±1.0			0.340
ESR (mm/h)			50.8±21.0	34.3±18.3			0.030

In the first stage, dynamic spacers were placed in 23 patients and static spacers in 21 patients. These two groups were compared in terms of duration of antibiotic use, ROM, and C&F KSS after revision arthroplasty. The dynamic spacer group had a shorter antibiotic use duration and a higher effect on the ROM and C&F KSS results. However, there was no statistically significant difference (p = 0.676, p = 0.894, p = 0.551, and p = 0.941, respectively) (Table 5).

**Table 5:** The effect of spacer type on ROM and C&S KSS values. ROM: Range of motion; KSS: Knee Society Score. p<0.05 was considered statistically significant.

	Spacer type		
	Dynamic (n:23)	Static (n:21)	P-value
	Mean+SD	Mean+SD	
Duration of antibiotic use (week)	7.9±3.3	8.3±4.0	0.676
ROM (˚)	89.8±8.7	89.2±20.0	0.894
KSS Clinical	77.2±9.5	75.2±12.3	0.551
KSS Functional	64.1±17.4	64.52±17.9	0.941

Teicoplanin was added to the cement in 35 patients and imipenem in nine patients. The effect of antibiotic type on the duration of antibiotic use between the two stages was comparatively analyzed. The teicoplanin-containing cement group (7.6±3.1 weeks) had an earlier response to infection treatment than the imipenem-containing group (10.5±5.1 weeks), with a significant difference (p=0.041) (Table 6).

**Table 6:** Comparison of the effects of adding teicoplanin or imipenem to bone cement on the duration of antibiotic use. Values are mean±S.D. p<0.05 was considered statistically significant.

	Antibiotic type			
	Teicoplanin (n:35)	Imipenem (n:8)		P-value
Duration of antibiotic use (w)	7.6±3.1		10.5±5.1	0.041

The mean ROM value was 64.9 ± 13.8˚ (range: 0˚-87˚) before treatment and 89.5 ± 15.0˚ (range 45-120˚) after revision arthroplasty (p <0.001). The mean clinical KSS was 49.1 ± 10.3 (range: 29-65) before the treatment and increased to 76.3 ± 10.8 (range: 46-94) at the end of follow-up. Considering the change in functional KSS scores, the reported score was 36.8 ± 15.2 (range: 0-60) before the treatment and 64.3 ± 17.4 (range: 20-90) after the follow-up. There was a statistically significant difference between the pre-treatment and post-revision C&F KSS (p <0.001). Complications were observed in eight (18.2%) of the 44 patients during follow-up. These complications consisted of re-infection in five patients (10.4%), knee instability in one patient (2.1%), aseptic loosening in one patient (2.1%), and joint stiffness in one patient (2.1%). Patients who developed re-infection underwent two-stage revision arthroplasty. Insert exchange with a bigger size was performed in the 90-year-old patient with knee instability who was not very active in daily life. Physical therapy exercises were planned for the patient with joint stiffness. According to these results, the success rate of the treatment was 81.8%, and the rate of infection eradication was 89.6%.

## Discussion

The ultimate goal of the revision procedure is to eradicate the infection and reconstruct a functional, painless, and stable knee joint. Two-stage revision knee arthroplasty, with an infection eradication rate ranging from 54% to 100% (average rate of 84.8%), has demonstrated satisfactory outcomes for both patients and orthopedic surgeons [10]. The 89.6% infection eradication rate and 81.8% success rate achieved in our study support the literature in light of available information. High levels of serum biomarkers CRP and ESR play a critical role in the preoperative diagnosis of PJI. In addition, these two parameters are also used in the follow-up of infection treatment. Moreover, deciding the appropriate time for reimplantation in PJI has been a challenge for orthopedic surgeons, and the impact of CRP and ESR levels during the treatment process on post-treatment complications has been the subject of many studies. It was emphasized that serum CRP and ESR values had poor diagnostic value in facilitating the decision of the time of reimplantation [11]. It was shown that the percent change in CRP and ESR levels before the first stage and revision had no diagnostic effect on the timing of reimplantation [12]. However, these parameters do not have sufficient sensitivity and specificity, and an abnormal level of CRP or ESR before revision increases the possibility of reoperation after revision [13]. The findings from this study indicate that the changes in CRP and ESR levels were significant from the moment the infection was confirmed through to the successful treatment of the infection and into the post-revision period. One of the controversial issues is culture-negative PJI. As is known, identifying the causative microorganism, particularly from culture samples obtained intraoperatively during the first stage, holds paramount importance in determining the appropriate antibiotic therapy for effective infection eradication. Approximately 65-94% of cultures yield positive results [14,15]. On the other hand, it was reported that the prevalence of negative cultures in PJIs varied between 7 and 42% [16]. Therefore, surgeons still have doubts in determining the efficacy of treatment during the diagnostic process, in the second stage, and afterward, in case of negative cultures. In addition, the challenges in identifying infection-free survival can be considered another disadvantage in culture-negative cases. In contrast, there was no difference between culture-negative and culture-positive patients regarding treatment outcomes [17,18]. The prevalence of the results that were obtained from the culture samples in this study was consistent with the literature. In culture-positive patients, shorter systemic antibiotic therapy showed that the detection of the causative microorganism positively impacted the treatment protocol. Managing culture-negative PJI requires administering broad-spectrum antibiotics or multiple antibiotics against the most common infectious organisms. However, this approach places patients at risk of systemic toxicity and may not provide coverage against fungi or rare organisms. In addition, it is crucial to consider fungal PJIs, particularly in culture-negative patients with immunosuppressive co-morbidities. The incidence of fungal PJIs has been on the rise, and microbiological studies should be performed for these organisms also in order to identify the causative microorganism. Effectively managing fungal PJIs is as challenging as diagnosing them, necessitating a multidisciplinary treatment approach [19]. There is consensus among most surgeons regarding the addition of antibiotics to cement. However, there are still conflicts regarding the type and amount of antibiotics used. It was emphasized that to preserve the cement's mechanical properties, the antibiotic dosage should not exceed 4g per 40g of cement [20]. Local teicoplanin has better-sustained diffusion into the infected tissues and fewer adverse effects. Teicoplanin was reported to be a more effective option in two-stage revision arthroplasty, and it has been shown to be superior for infections caused by Staphylococcus aureus [21]. Teicoplanin-loaded cement showed better elution efficacy and provided longer inhibitory periods against methicillin-sensitive Staphylococcus aureus (MSSA) and methicillin-resistant Staphylococcus aureus (MRSA) than cement loaded with vancomycin or daptomycin [22]. Teicoplanin has synergistic or additive activity with many other antibiotics, which are used for systemic antibiotherapy and shortens the duration of the systemic antibiotherpy. A total of 57% of our patients received antibiotherapy in less than six weeks, and none of our patients needed antibiotherapy for more than 20 weeks. A shorter course of IV antibiotic therapy decreases systemic toxicity and may reduce the emergence of drug-resistant organisms. Imipenem provides coverage against Gram-positive and Gram-negative microorganisms as well as against Pseudomonas. In the present study, there was a significant difference in favor of teicoplanin in terms of the duration of antibiotic use between the two stages compared to imipenem. However, the outcomes of both antibiotics were similarly satisfying after revision, which supports the literature.

Another aim of two-stage revision knee arthroplasty is to obtain a functional joint. For this purpose, the joint space is protected by spacers placed during the first stage. Current studies showed that dynamic spacer use had higher ROM and clinical scores than static use at the end of follow-up. However, there was no significant difference between dynamic and static spacers regarding clinical scores and complications [23,24]. In addition, the use of dynamic spacers improves patients' quality of life between the two stages and increases patient compliance [25,26]. In this study, the ROM and C&F KSS values after treatment were higher in patients with dynamic spacers.

The time interval between the two stages and the optimal timing of revision arthroplasty remains controversial. While maintaining a short time interval between the two stages increases the possibility of a failed infection treatment, a longer time interval leads to an increase in muscle atrophy, a decrease in bone mineralization and complicates the rehabilitation process after revision surgery. In addition, other disadvantages of maintaining a longer time interval between the two stages include higher treatment costs and reduced patient satisfaction [27]. We prefer prolonged administration of antibiotics (>6 weeks) if the patient is immunocompromised, the soft-tissue envelope is poor, a large draining sinus is present, or the infecting organism is MRSA. Previous studies have indicated that the time interval between stages should be at least six weeks [27,28]. According to Bernard L et al., total post-surgical treatment for six weeks was equivalent to 12 weeks in terms of cure. Early switching to oral antibiotics is as effective as prolonged parenteral regimens. However, this approach requires confirmation in randomized trials [28]. Vielgut I et al. reported that performing revision arthroplasty within 12 weeks resulted in infection eradication at a rate of 90% [29]. In this study, the mean time interval was quite long, largely due to the prolonged antibiotic therapy administered to 10 patients with large drainage fistulas and three patients older than 80 years who were not medically fit to undergo a two-stage procedure within a short time interval. ROM was found to be significantly higher at the end of the follow-up period in patients who received treatment within intervals of 10 weeks.

The present study has some limitations. It was not a randomized retrospective study, and the relatively small number of patients limited the ability to generalize the findings. Future randomized studies that include a larger patient population and provide long-term results could potentially yield more promising outcomes.

## Conclusions

A significant decline in CRP and ESR levels may be considered an indication that it is safe to proceed with reimplantation surgery. The duration between the two stages, with a maximum limit of 10 weeks, appears to have a positive impact on ROM after revision surgery. Whether a dynamic or static spacer was used before reimplantation did not have a statistically significant effect on clinical outcomes. The addition of teicoplanin to bone cement seems to shorten the duration of infection treatment. Furthermore, patients who were culture-negative and those who were culture-positive exhibited similar clinical scores.
